# A transformer model for learning spatiotemporal contextual representation in fMRI data

**DOI:** 10.1162/netn_a_00281

**Published:** 2023-01-01

**Authors:** Nima Asadi, Ingrid R. Olson, Zoran Obradovic

**Affiliations:** Department of Computer and Information Sciences, College of Science and Technology, Temple University, Philadelphia, PA, USA; Department of Psychology and Neuroscience, College of Liberal Arts, Temple University, Philadelphia, PA, USA; Decision Neuroscience, College of Liberal Arts, Temple University, Philadelphia, PA, USA

**Keywords:** Dynamic functional connectivity, Transformer models, Attention mechanism, Graph convolution networks, Feature learning, Deep learning

## Abstract

Representation learning is a core component in data-driven modeling of various complex phenomena. Learning a contextually informative representation can especially benefit the analysis of fMRI data because of the complexities and dynamic dependencies present in such datasets. In this work, we propose a framework based on transformer models to learn an embedding of the fMRI data by taking the spatiotemporal contextual information in the data into account. This approach takes the multivariate BOLD time series of the regions of the brain as well as their functional connectivity network simultaneously as the input to create a set of meaningful features that can in turn be used in various downstream tasks such as classification, feature extraction, and statistical analysis. The proposed spatiotemporal framework uses the attention mechanism as well as the graph convolution neural network to jointly inject the contextual information regarding the dynamics in time series data and their connectivity into the representation. We demonstrate the benefits of this framework by applying it to two resting-state fMRI datasets, and provide further discussion on various aspects and advantages of it over a number of other commonly adopted architectures.

## INTRODUCTION

Analysis and modeling of brain’s blood oxygen level–dependent (BOLD) activity and functional connectivity (FC) through functional magnetic resonance imaging (fMRI) have led to utilization of an expanding array of methodological tools such as graph theory, machine learning, and statistical tests (Bastos & Schoffelen, 2016; Y. He & Evans, 2010; Rogers, Morgan, Newton, & Gore, 2007). A powerful class of machine learning approaches for building predictive models is the deep architectures of artificial neural networks, also known as deep learning models (Deng & Yu, 2014; LeCun, Bengio, & Hinton, 2015). Deep learning models are able to capture higher level nonlinearities and to learn informative representations in order to facilitate training a multitude of modeling tasks with little to no requirement for feature selection (LeCun et al., 2015). This family of predictive models has proven to be a powerful tool for a diverse set of analytical tasks, including feature selection, pattern discovery, feature learning, and predictive modeling (Sarraf & Tofighi, 2016a; Wen et al., 2018; Yin, Li, & Wu, 2022).

Several deep learning architectures have been utilized recently to analyze fMRI data in areas such as predictive modeling, representation learning, and adversarial data augmentation and synthesis (Dado et al., 2022; Dong et al., 2020; Frolov, Maksimenko, Lüttjohann, Koronovskii, & Hramov, 2019; Kawahara et al., 2017; J.-H. Kim et al., 2021; Li, Satterthwaite, & Fan, 2018; Riaz, Asad, Alonso, & Slabaugh, 2020; Sarraf & Tofighi, 2016b; Suk, Wee, Lee, & Shen, 2016; Zhuang, Schwing, & Koyejo, 2019).

An important factor in deep learning’s superior performance is its ability in learning an effective representation from the data to facilitate the task of predictive modeling. One of the main objectives of representation learning (also known as feature learning) is informative encoding of the input data; this encoding embeds hidden dependencies and patterns of the data into the learned features to serve several downstream tasks such as regression, classification, imputation, and forecasting (Liu et al., 2015; Zerveas, Jayaraman, Patel, Bhamidipaty, & Eickhoff, 2021). Encoding has gained significant attention in recent years for disentangling latent characteristics in data in various applications with limited supervision. A representation’s advantage relies on its power in capturing the information from a broad set of characteristics and contextual knowledge in the data (Bengio, Courville, & Vincent, 2013). Therefore, in the field of fMRI data analysis, learning a conclusive representation requires obtaining not only the contextual information regarding spatial dependencies but also the variations in connectivity topology through the course of the fMRI experiment. Dynamic functional connectivity (dFC) of the brain is generally highly volatile because of variables such as cognitive tasks and states, as well as spontaneous fluctuations in resting-state BOLD signal, either in normal conditions or during sleep and different levels of anesthesia (Chen, Nomi, Uddin, Duan, & Chen, 2017; Chou et al., 2017; Mantini, Perrucci, Del Gratta, Romani, & Corbetta, 2007). Static functional connectivity analysis fails to capture such dynamics that characterize the interactions and contexts between the activities of different regions of the brain. Therefore, true modeling of functional connectivity requires dynamically capturing time-dependent aspects of spatial dependencies. Popular architectures such as convolutional neural networks (CNNs), recurrent neural networks (RNNs), and long short-term memory (LSTMs) have been employed for the modeling fMRI data. However, these architectures suffer from certain shortcomings when dealing with large-scale evolutionary graphs (Scarselli, Gori, Tsoi, Hagenbuchner, & Monfardini, 2008; Wan et al., 2019). These disadvantages include, but are not limited to, lack of true contextual modeling and adaptability with graph’s flexible topology, the inability in preserving information over longer graph “walks,” and inefficient training time. These shortcomings are addressed by a recently popular architecture called the transformer (Vaswani et al., 2017). The transformer is a powerful deep learning model that confers the context for any position in the input sequence by adopting an attention mechanism while facilitating efficient parallel training (Vaswani et al., 2017; Wolf et al., 2020; Zerveas et al., 2021). Because of these capabilities, this class of deep learning models has rapidly become the dominant architecture in many complex machine learning tasks and has proven to be adaptable to various structures such as graphs and time series to learn spatial, temporal, and positional context in the data (T. H. Kim, Sajjadi, Hirsch, & Schölkopf, 2018; Plizzari, Cannici, & Matteucci, 2021; M. Xu et al., 2020; C. Yu, Ma, Ren, Zhao, & Yi, 2020). The attention mechanism is one of the main frontiers in representation learning, which was developed to enhance the encoder-decoder performance on long input sequences. The core idea behind attention on sequence data is that instead of relying merely on the context vector, the decoder also uses the past states and time steps of the encoder. The attention weights are therefore introduced as trainable parameters that assign different importance levels to the different elements of the input sequence. The advantages of attention is its capability in identifying the information in an input element that is most pertinent to carrying out a prediction task with high accuracy (Vaswani et al., 2017; Wolf et al., 2020).

Inspired by the proposed transformer models for various applications in recent years, in this work we adopt a framework for jointly learning the embedding of spatiotemporal contextual information within fMRI data based on a transformer architecture that utilizes the concepts of attention mechanism as well as graph convolution network. The objective of the proposed framework is to learn a set of embedded features that capture a holistic representation regarding the dynamics and dependencies within the fMRI data. For this purpose, the proposed model leverages both the multivariate BOLD time series and the dFC networks simultaneously to learn a representation that takes into account the spatial and temporal contextual relations within both of the mentioned input data components. The extracted representation can then be used in several applications such as classification between cohorts of data, anomaly detection in activation patterns, and feature selection. In this work, the derived contextual representations are utilized for classification tasks and are compared with several commonly used baseline models for assessment. For this purpose, we put forward two binary classification tasks where the model is trained to predict subjects diagnosed with autism spectrum disorder (ASD) from healthy subjects in one task, and the sex of the subjects in the second task.

In the next section we discuss the different building blocks of the proposed framework, followed by experimental results. We then discuss the advantages and shortcomings of the proposed approach in the discussions.

## METHODOLOGY

In this section, we describe the proposed spatiotemporal transformer framework for representation learning and modeling of activity and dFC of brain’s regions. We first lay out the task of modeling dFC as a classification problem, and then explain the overall architecture of the transformer framework. Afterwards, we describe each building block of the proposed approach in detail. The definitions of the terminologies used in this section are provided in the margin.

### Problem Formulation

Dynamic functional connectivity of the brain can be represented as an evolving graph characterized by varying intensity of interactions between its regions. The dFC network is composed of separate regions of the brain as the nodes, and their coactivation over a temporal window as the weight of the links connecting them. We express this graph as *G* = (*V*, *E*, *T*), where *V* = {*v*_1_, *v*_2_, …, *v*_*N*_} is the set of *N* vertices, *E* is the set of edges, and *T* = {*t*_1_, *t*_2_, …, *t*_*τ*_} is the set of *τ* time steps of the experiment during which the dFC graph *G* evolves. To learn the higher order spatiotemporal representation of dependencies in the dFC network, we formulate the model’s training process as a classification problem with the objective of distinguishing between cohorts of subjects. Through the training process, the weights within the different blocks of the transformer are learned, and the trained model generates the representation of spatiotemporal dependencies *S*_*t*_*i*__ as a vector of features for each node *v*_*i*_ at time step *t*. To learn this new set of features, the transformer leverages the BOLD time series of the brain regions as well as their dFC networks within each temporal window *t*_*w*_ simultaneously. The time series are utilized by an attention mechanism to extract the spatial and temporal context for each node *v*_*i*_ at time step *t* ∈ *t*_*w*_, and the functional connectivity network within *t*_*w*_ is adopted by a graph convolution network (GCN) to inject the topological information of connectivity into the newly generated features (Gadgil et al., 2020; Kipf & Welling, 2016; Wang, Li, & Hu, 2021). The output of the two embedding units are then fused together to provide a rich set of features with spatiotemporal contextual knowledge of the data, which can in turn facilitate analysis and prediction tasks. This model can be applied on different spatial resolutions including training on specific regions of interest (ROIs) where the nodes are the voxels within the regions, or at a lower resolution setup where each ROI constitutes a node.

In the following sections we explain in detail the architecture for spatiotemporal representation learning based on the time series and the dFC network. We then lay out the details of the experimental setup in the Results section.

### Overall Architecture

**Figure 1.**
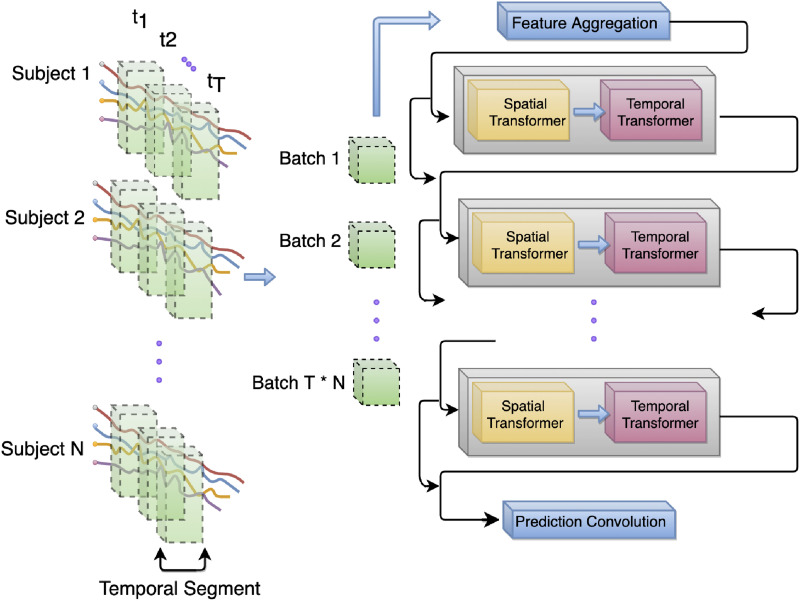
Overall architecture of the transformer model and input batch data preparation. Each block in gray color is a spatiotemporal (ST) block containing one spatial and one temporal transformer.

To learn the higher order representation of dynamic spatiotemporal dependencies, we develop a two-tier architecture that includes a spatial transformer followed by a temporal transformer. The general schema of this approach is provided in Figure 1, where the spatial and temporal components are placed sequentially within each spatiotemporal (ST) block (the blocks in gray). The ST blocks are also positioned sequentially, meaning that the output of the temporal component of each ST block is used as the input to the spatial transformer of the next ST block, except the final ST block, where the output of the temporal component is supplied to the prediction layer. The input to the first ST block (i.e., the spatial component of the first ST block) is a positional embedding of the time series data within the temporal window *t*_*w*_ based on a 1 × 1 convolution layer, as well as the dFC network constructed based on the coactivations of the BOLD time series within *t*_*w*_. As depicted in Figure 1, the input of the next spatial blocks include the embedding of the features that are the output of the previous temporal block, aggregated with the input to the previous block, as well as the dFC network constructed based on the coactivations within *t*_*w*_. The input-output aggregation, also known as residual connection, is widely adopted in deep learning architectures because of its advantage in providing a stable training and enhanced representation in each block (K. He, Zhang, Ren, & Sun, 2016; Jastrzebski et al., 2017). We also adopt the residual connections within each spatial and temporal transformer because of the same advantages.

The sequential training process is performed for every batch of time series data until the model converges based on the assigned error metric. In the next step, we describe the batch data preparation process for training our transformer model.

### Batch Data Preparation

After preprocessing the BOLD time series and generating the dFC networks within each temporal window, batch data preparation is needed in order to facilitate the training process by the transformer model. This is because large models such as transformers require large input data for robust training, as otherwise the weights and hidden features remain underdetermined. In order to create batches of input data, the time series for each region of interest are sliced according to a fixed window size *T*_*τ*_ with temporal overlap *T*_*ϕ*_. In other words, instead of using the entire time series $d_{i}^{S}$ of each voxel $v_{i}^{S}$ for each subject *S* as the input data (i.e., *S* input data points for *S* subjects), *M* segments of each time series are used as the inputs, resulting in an adequately larger dataset (*S* × *M* input data points for *S* subjects) and robust training of the transformer model. This process is depicted in the left side of Figure 1. For this study, we selected the window size *T*_*τ*_ = 25, and temporal overlap *T*_*ϕ*_ = 5 as the default setup of our analysis on the first dataset, and *T*_*τ*_ = 50, and temporal overlap *T*_*ϕ*_ = 10 for the second experimental dataset. This preparatory step resulted in 15,000 time series slices for each voxel *v*_*i*_ for the first dataset and 31,680 segments for each voxel for the second dataset. The details of the datasets used in this study will be discussed in the Results section along with an analysis of the effect of temporal window size on the classification performance.

We also set the size of each input batch to 50 entries, where each entry is composed of two components: the multivariate time series segments of the temporal window *t*_*w*_ for the *N* voxels within the ROI, as well as the FC adjacency matrix based on the coactivations of the same time series segments. The prepared input batches are then supplied to the first ST block to begin the process of training.

### Spatial Transformer

**Figure 2.**
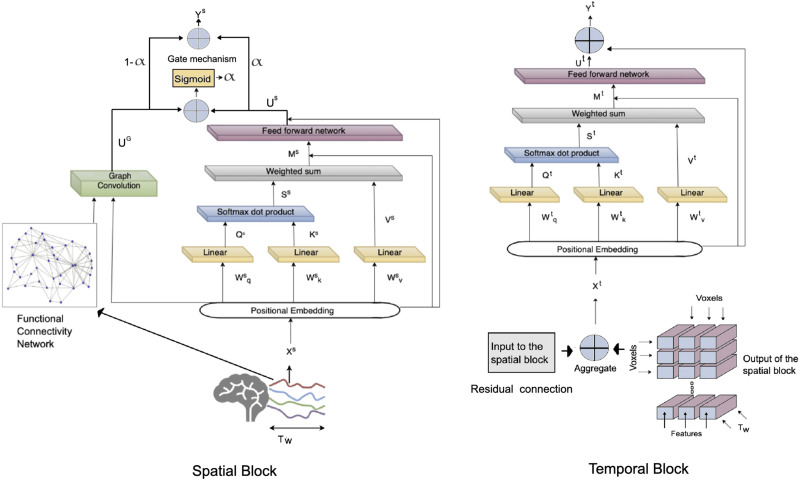
Block-level architecture of the transformer model. Left: The architecture of the spatial transformer component, where *T*_*w*_ is a temporal window (time series segment) within which the input data are derived, and *y*^*s*^ is the output of this transformer. The output of the positional embedding is supplied to the graph convolution network and the attention in parallel. The output of these two components is then fused through a gate mechanism to generate the features. Right: The architecture of the temporal transformer block. The input to this block is the output of the spatial block combined with the input to the spatial block by a residual connection (also see Figure 1).

The spatial transformer consists of a spatial positional embedding layer that provides the encoding for the attention mechanism, a dynamic graph attention layer to inject the spatial context of each node’s BOLD activation level into the newly generated features, and a GCN to embed the topological properties of the FC network within *t*_*w*_. The building blocks of the spatial transformer are depicted in Figure 2, where the output of positional embedding is supplied to the attention and GCN blocks simultaneously. We explain each block of the spatial transformer in the following sections.

#### Positional embedding.

An embedding of the time series data is needed to introduce the positional information of each node to the attention block. For this purpose, a 1 × 1 convolution layer is adopted to encode the positional features into a *d*-dimensional vector for each node at each time step, where *d* is the embedding size. For spatial positional embedding, we adopt the approach proposed by Wang et al. (2021), in which functional connectomic neighborhoods are used as the topological input through the adjacency matrix of the functional connectivity network. For temporal positional encoding, trigonometry-based feature transformation was performed by calculating the sine and cosine values of each time step and using them as the temporal embedding of each time series value. The benefit of this approach over one-hot encoding of temporal features is that it avoids generating a high-dimensional and unbalanced vector of positional encoding features. The 1-D depthwise convolution is then used to convert the positional information into a feature vector of appropriate size for each node at each time step *t* (Mandal & Mahto, 2019; Vosoughi, Vijayaraghavan, & Roy, 2016). This block outputs a vector for each node at each time step *t* ∈ *t*_*w*_, containing the spatial and temporal information that is in turn used by both the GCN and the dynamic graph attention blocks in parallel, as depicted in Figure 2.

#### Graph convolution block.

Graph convolution network is a variant of convolutional neural networks (CNN); it learns a representation of graphs by leveraging their structure and aggregate node information from its neighborhood in a convolutional fashion. To learn the structure-aware node features based on the connectivity topology, a convolution approximated by Chebyshev polynomials is employed (Defferrard, Bresson, & Vandergheynst, 2016). A GCN setup for classification task on resting-state fMRI was suggested by Wang et al. (2021) in which the functional connectivity network is used instead of the network of Euclidean distances as the topological input to facilitate an encoding that is appropriate for the organization of the brain. We build upon this approach by using the FC network for the GCN in parallel with the attention mechanism within the spatial component. However, a difference between our proposed setup and the setup proposed by Wang et al. is that they adopt the time series of the nodes as input features, whereas we utilize the embedded features of the nodes (from the previous block) within each time *t* ∈ *t*_*w*_ as the input features to GCN, as depicted in Figure 2. Therefore, the input to the GCN includes the embedding of the time series segments from the previous block as the vector of features for each node at each time *t* ∈ *t*_*w*_, as well as the functional connectivity of the same time series segments as the network input. The GCN mechanism first aggregates all the features of the neighbors of every node, including itself, through an aggregate function. The aggregated feature sets are then passed through a nonlinear neural network layer to output a vector of features for each node at every time point. This vector is finally fused together with the results of the dynamic attention layer via a gate mechanism to create the output of the spatial block, as depicted in Figure 2.

#### Dynamic attention block.

To capture the contextual time-evolving functional dependencies between the nodes, we adopt a dynamical graph attention mechanism that maps the embedded features of each node $\tilde{X}$ from the positional embedding block to high-dimensional latent subspaces. Attention mechanism consists of three main components: query, key, and value (Vaswani et al., 2017). The set of input vectors that we aim to calculate the attention for is called a query, and the set of vectors to calculate attention against is called the key. For each query, the similarity between the query and the keys is calculated, which provides a score for each key-query pair. In this study a dot product attention is adopted, meaning that it calculates the inner product between the query and a key vector to provide the similarity score between them (Vaswani et al., 2017). This process can be performed for multiple key, query, and value vectors at once; therefore, packing together sets of queries, keys, and values, we have the *Q*^*S*^, *K*^*S*^, and *V*^*S*^, such that

$$\begin{array}{l} Q^{S}={\tilde{X}}^{S}W_{Q}^{S},\\ K^{S}={\tilde{X}}^{S}W_{K}^{S},\\ V^{S}={\tilde{X}}^{S}W_{V}^{S}, \end{array}$$ (1)

where $W_{Q}^{S}$, $W_{K}^{S}$, and $W_{V}^{S}$ are the projection matrices that are used to generate the subspace representations of the query, key, and value matrices. Each row of *Q*, *K*, and *V* represents an entity, therefore the dot product attention takes a weighted sum of the entity values in *V* where the weights are given by the interactions of query-key pairs. This process is depicted in Figure 2, where the dynamic spatial dependencies calculated from the query-key dot product is then supplied to a softmax function for scaling, and then multiplied with the value matrix *V*^*S*^ to update the node features.

$$\text{Attn}\left(Q,K,V\right)=\text{softmax}\left(\frac{{QK}^{S}}{\sqrt{d^{S}}}\right)V^{S},$$ (2)

where *d* is the feature dimension. As the next step of the spatial component, a three-layer feed-forward neural network with nonlinear activation is applied on each node’s weighted sum contextual features to capture the interactions between the features, as in Vaswani et al. (2017).

$$U^{S}=\text{ReLU}\left(\text{ReLU}\left(\text{Attn}\left(Q,K,V\right)W_{1}^{S}\right)W_{2}^{S}\right)W_{3}^{S},$$ (3)

where $W_{i}^{S}$ is the weight matrix for the *i*th layer and ReLU stands for rectified linear unit.

**Figure 3.**
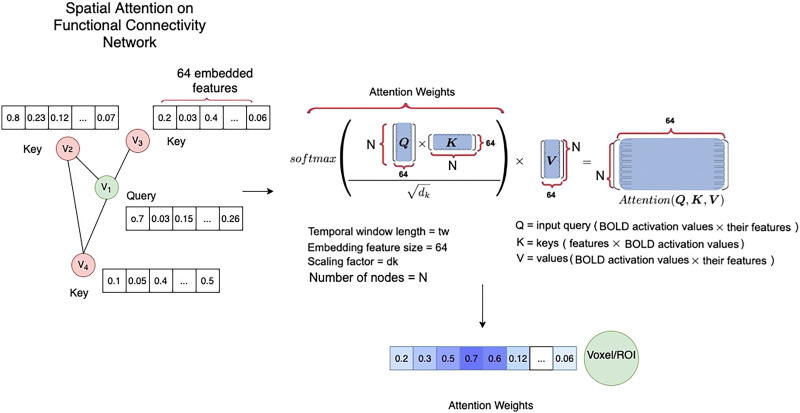
The attention mechanism within the spatial block. Similarity between the features of each voxel (query) and other voxels (keys) within the FC network is calculated through the dot product process and is reweighted during the training process to create the attention weights for the input sequence.

This process is illustrated in Figure 3, where four example nodes (voxels or regions of interest, depending on spatial precision) constitute the functional connectivity network. The query node in this figure is voxel *V*1, and each node is assigned a feature vector, which is the output of the positional encoding on the time series prior to the attention block. As this figure demonstrates, the similarity between the query node and every other node (keys) is obtained through the dot product of its encoded features, which divided by a scaling factor (see Equation 7) provides the attention weights for the nodes. The attention weights emphasize parts of the FC network while diminishing other parts based on their contextual importance for the prediction task. For voxel *v*_1_ as the query, the output vector *Y*_1_ is derived by

$$W_{11}v_{1}^{f}+W_{12}v_{2}^{f}+W_{13}v_{3}^{f}+\ldots +W_{1N}v_{N}^{f}=Y_{1},$$ (4)

where $v_{i}^{f}$ are the input feature vectors for voxels *v*_*i*_, and *W*_1*i*_ correspond to the attention weights based on similarity of features between voxel *v*_1_ and every other voxel. This process is performed for every node in the network, meaning that each node plays the role of the query separately. Thus, for each node at time step *t*, the input to the attention mechanism is a vector of its features, and the output consists of a vector with contextual information. The weights of query, key, and value layers are then updated through back-propagation during training. Therefore, through the spatial attention process, the context of the nodes (voxel/ROI) with regard to the other nodes within the FC network at time *t* is extracted to be combined with the output of the GCN block to form the spatial representations.

The last step of the spatial component is the gate mechanism, which is applied to fuse the spatial features learned from the GCN and the dynamic attention block. The steps of the gate mechanism include aggregating the features from GCN and attention block, calculating the sigmoid of this aggregation, and then using the sigmoid output to create a weighted sum of the output of GCN and attention block such that

$$Y^{S}=\alpha U^{S}+\left(1-\alpha \right)U^{G}.$$ (5)

The output of this operation is a vector of features for each node at each time step *t* ∈ *t*_*w*_. Therefore, for *N*_*f*_ number of features, *t* time steps within the temporal window *t*_*w*_, *N* nodes, and a batch size *N*_*b*_, the output of the spatial block is a tensor of size *N*_*b*_ × *t* × *N* × *N*_*f*_. This output is then supplied to the temporal transformer component of the ST block, as illustrated in Figure 2. In the next part, we explain the building blocks of the temporal transformer.

### Temporal Transformer

Left-to-right architectures of temporal dependencies such as RNN models are limited to consider temporal dependencies based on preceding time steps, and fail to consider contextual dependencies. Therefore, for the temporal transformer we also adopt a attention mechanism to incorporate the temporal information, similar to the spatial transformer. The input to the temporal component is the embedded features, which are obtained by passing the concatenation of the input features *X*^*s*^ aggregated with the temporal embedding *X*^*T*^ (i.e., the output of the previous spatial block and its input as the residual connection). Similar to the spatial transformer, this input is passed to a 1 × 1 convolution positional embedding layer:

$$X^{T}={\text{Conv}}_{t}\left(X^{T},D^{T}\right),$$ (6)

where *X*^*T*^ = *X*^*S*^ + *Y*^*S*^ is calculated from the outputs of the spatial transformer block, and *D*^*T*^ is the temporal embedding. Therefore, we obtain an embedding of features as a vector for each node at each time step *t* within the temporal window *t*_*w*_. Similar to the spatial transformer, we have

$$\begin{array}{l} Q^{T}={\tilde{X}}^{T}W_{Q}^{T},\\ K^{T}={\tilde{X}}^{T}W_{K}^{T},\\ V^{T}={\tilde{X}}^{T}W_{V}^{T}, \end{array}$$ (7)

where $W_{Q}^{T}$, $W_{K}^{T}$, and $W_{V}^{T}$ are the learned liner mappings. Here we also adopt the scaled dot product function to consider bidirectional temporal dependencies.

$$\text{Attn}\left(Q,K,V\right)=\text{softmax}\left(\frac{Q^{T}K^{T}}{\sqrt{d}}\right)V^{T}.$$ (8)

**Figure 4.**
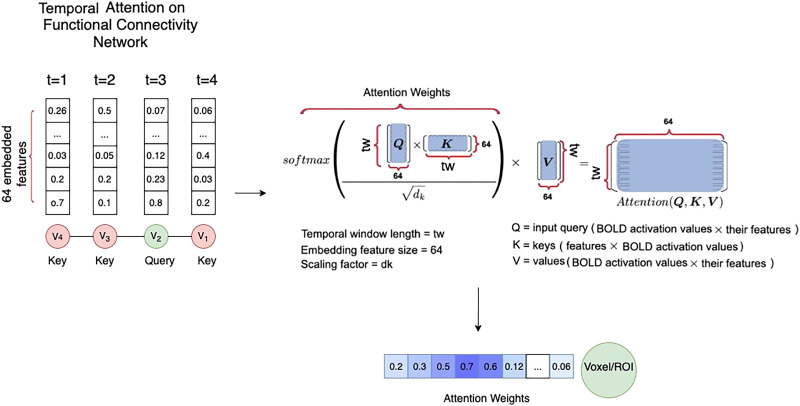
The attention mechanism within the temporal block. Similarity between the features of each voxel at time *t* (query) and its own values on other time steps (keys) is calculated through the dot product process and is reweighted during the training process to create the attention weights for the input sequence.

Then, to explore the interactions among latent features, a shared three-layer feed-forward neural network is developed whose output is aggregated with the output of positional embedding unit as a residual connection to create the vector of features for each node for time step *t* within *t*_*w*_, as depicted in Figure 2. Unless the temporal transformer belongs to the final ST block, the aggregation of its output *Y*^*t*^ with its input *X*^*t*^ is supplied to the spatial block of the next ST block. However, if the temporal transformer is a part of the final ST block, its output is supplied to the prediction layer. This procedure is depicted in Figure 4, where the dot product is calculated between the feature vector for each query node at time step *t*_*i*_ with the features of the same node at other time steps. Aside from this difference between the temporal and spatial attention, the rest of the process for capturing the contextual vector for each node is similar. Thus, the output vector for voxel *v* at time point *t* is obtained from the following equation:

$$W_{t1}v_{t=1}^{f}+W_{t2}v_{t=2}^{f}+W_{t3}v_{t=3}^{f}+\ldots +W_{t\tau }v_{t=\tau }^{f}=Y_{t},$$ (9)

where $v_{t=i}^{f}$ are the input feature vectors for voxel *v* at time step *i*, and *W*_*ti*_ corresponds to the attention weights based on similarity of features between voxel *v*_1_ at time *t* and its features at time *i*. Therefore, in the temporal attention block, the attention weights enhance parts of the time series sequence while diminishing other parts based on their contextual importance for the prediction task. In the next section, we discuss the prediction layer as a unit outside of the ST blocks.

### Prediction Layer

The prediction layer consists of two fully connected convolution layers with a ReLU activation function in between, which is similar to the feed-forward network used in Vaswani et al. (2017), followed by a softmax activation function for classification. This architecture for prediction layer has been commonly adopted to introduce nonlinearity that assists the model with learning complex mappings between the inputs and target variables (Agarap, 2018; Ide & Kurita, 2017).

The encoder component of the transformer generates a set of embedded features for each node at each time step. Consequently, the input to the prediction layer is a batch of size *N*_*b*_ of three-dimensional tensor of *N*_*f*_ spatiotemporal features yielded from the final ST block for each node *N* at each time point *t* ∈ *t*_*w*_. The output of this layer is a prediction depending on the downstream task. For classification tasks, the AUC was measured through cross-entropy between the predicted labels and the true labels. In the next section we provide the experimental results based on the discussed transformer architecture.

### Training Setup

In this section we provide the details and parameters of data preparation and the experimental setup. The implementation code for the methodology is available in Python via https://github.com/ThisIsNima/Spatio-Temporal-Transformer (Asadi, 2022). All the experiments were performed on an Intel Core i7-3370 CPU, 3.40 GHz with 32 GB of RAM, and the implementation code was written in Python programming language. The average training time of the spatiotemporal transformer model for the ROI-level analysis on the Autism Brain Imaging Data Exchange (ABIDE) dataset was 22 min and 16 s, and for the HCP data it was 28 min and 32 s.

The segmentation process was performed on the preprocessed time series data with the window length *T*_*τ*_ = 25 and temporal overlap *T*_*ϕ*_ = 5 for the first dataset, and *T*_*τ*_ = 50, and overlap length *T*_*ϕ*_ = 10 for the second dataset. Batch size was set to *N*_*b*_ = 50 for both datasets. For training, validation, and testing, the data were selected randomly from this data subset for each ROI, and then the training group was partitioned into batches of 50 items. The FC networks were then generated for the time series of each data entry within each window *t*_*w*_. Therefore, each of the 50 entries within each input batch for a region of interest included the time series segments for its *N* voxels as well as their FC network. A positional embedding of the two data components is then derived through a 1 × 1 convolution on the spatial and temporal encodings of the time series data to output a vector of features for each node at time point *t* within *t*_*w*_. Therefore, the output of the positional embedding is a 4D tensor of size *N*_*b*_ × *N* × *t*_*τ*_ × *N*_*f*_, where *N*_*f*_ is the embedding feature size, which was set to 64 for this experiment. The vector of embedded features is then supplied to the dynamic attention unit, and the pair of time series embedding output and FC adjacency matrix are supplied to the GCN unit of the spatial transformer. The spatial and temporal components are placed sequentially to form a spatiotemporal block. Three spatiotemporal blocks with 2-head dot product attention mechanisms were adopted for this analysis. Also, the initial leaning rate is set to 10^−4^ with a decay at a rate of 0.5.

Two resting-state fMRI datasets were used as the case studies in this work. The first dataset for this study is composed of 600 subjects from the ABIDE database, including 300 subjects diagnosed with ASD and 300 control subjects (Di Martino et al., 2014). This dataset was preprocessed by the Configurable Pipeline for the Analysis of Connectomes (C-PAC) pipeline and was slice time and motion corrected (*MS Windows NT kernel description*, n.d.). Also, the voxel intensities were normalized through global signal regression. The automated anatomical labeling (AAL) atlas was then adopted for parcellation of regions of interest (Tzourio-Mazoyer et al., 2002). The BOLD time series were then segmented using the sliding-window approach, and Pearson’s correlation between the time series within each temporal window *t*_*w*_ was calculated to generate the weight of the links between the nodes. The second dataset was constructed from data provided by the Human Connectome Project (HCP S1200) release comprising 440 subjects (age range: 22–37, mean age: 28.7 years; 220 males), where male and female subjects were matched for age (Van Essen et al., 2013). The resting-state BOLD data comprised 1,200 functional volumes per subject, and the AAL atlas was also used for parcellation of regions of interest. The demographic characteristics of the two datasets are provided in Table 3 in the Supporting Information.

Two classification tasks were set up to evaluate the performance of the model based on the features generated on the two experimental datasets. The objective of the classification tasks was to assess the quality of the generated features for distinguishing between cohorts of subjects based on fMRI data. In other words, the aim of this analysis was to evaluate how well the generated features characterize the BOLD activation pattern of each region within the context of global spatiotemporal dynamics of the brain’s regions by taking the spatiotemporal context of its BOLD activation dynamics as well as the dFC networks into consideration. After training the transformer model, it is supplied with test data to distinguish between the ASD and control subjects for the first dataset (ABIDE), and predict the sex of the subjects for the second dataset (HCP). For both classification tasks, 70% of the dataset was used for training, 15% for cross-validation, and 15% for testing.

An analysis of the effects of various architectural configurations on model’s performance is provided in Figure 1 in the Supporting Information. In this analysis, we investigated the combination of three different values for the number of attention heads, the embedding feature size, and the number of ST blocks against the model’s average classification AUC on 10 trials for both datasets. This analysis was the basis for our configuration setup. Furthermore, the effect of various temporal window sizes on the model’s performance is explored in the next section.

The experiments were performed on two spatial resolution levels including voxel-level analysis, and ROI-level analysis. In voxel-level analysis, a model is trained for each region, and the voxels within the ROI represent the nodes of the graph, whereas in the ROI-level analysis, a model is trained on the entire brain, where the regions of interest play the roles of graph nodes. For the ROI-level analysis, the times series of the voxels within each region are averaged to create one time course per ROI.

In our voxel-level experiments, we trained the model for each region separately in parallel, and then used an ensemble majority voting criteria for the prediction step. This setup has the benefits of more localized representation learning by considering the biological properties of the regions independently, as well as significantly enhancing the computational efficiency. Moreover, quite similar to the general principle of bagging ensemble training approach, these criteria can reduce the variance of the model. Therefore, during test, the model trained on each region predicts the class label of the test data from the same region, and a simple majority voting among the regions is used to determine the final classification of the subject from the test data.

Two comparative experiments are designed to compare the predictive power of learned representations for each of the two experimental case studies. For the first set of experiments, we adopted a standalone GCN model that takes the time series positional embedding as well as the FC network as the input, a standalone attention block (SA) as the second baseline, and a feed-forward convolution neural network (FF-CNN) as the third baseline, where the latter two baselines use the spatiotemporal embedding of the time series data within each temporal window as the input. The reason for adopting the first two baselines was to compare how well each of the two blocks of our model performs as popular standalone architectures. To compare the performances, the area under the classification ROC curve (AUC) were compared on unseen test data. In the next section, we first provide example visualizations and preliminary analysis of the results, and then offer the results of the classification tasks. For the second comparative analysis, three deep learning–based models that are used for fMRI classification were used as baselines. These three models include spatiotemporal graph convolutional networks (ST-GCN), deep-fMRI, and the multiscale RNN (MsRNN; Gadgil et al., 2020; Kong et al., 2021; Riaz et al., 2020; Yan et al., 2019; B. Yu, Yin, & Zhu, 2017).

## RESULTS

In this section, we discuss the experimental results based on the proposed architecture on two sets of resting-state fMRI datasets discussed in the Methodology section. We first provide a preliminary analysis of the representations, including visualizations of the attention maps of number of brain regions, and then provide the classification results. For region-specific voxel-level analysis, we provide the visualizations for four regions, namely left and right amygdalas and hippocampus in this section, and the results for other regions in Table 4 of the Supporting Information. The importance of the four mentioned regions in understanding memory and analysis of ASD and other neurological conditions according to related literature is the factor in choosing these regions for the visualizations (Burgess, Maguire, & O’Keefe, 2002; Guo et al., 2016; Treves & Rolls, 1994; Q. Xu, Zuo, Liao, Long, & Wang, 2020). Furthermore, we provide the visualization for the the ROI-level full-brain analysis in this section.

### Analysis of the Representations

**Figure 5.**
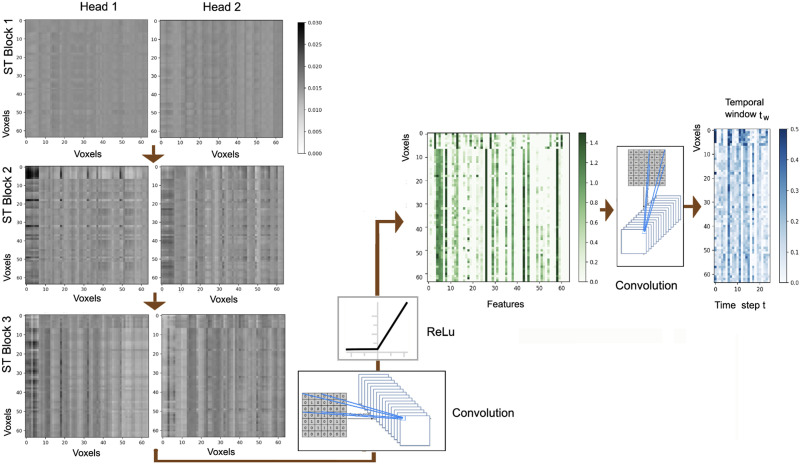
A visualization of the attention maps based on each transformer head and prediction block for the left amygdala (region 41 per AAL atlas) of one subject for the first temporal window, where the window size is 25 time steps, and the embedding feature size is 64.

A visualization of the outputs of the ST blocks for the left amygdala of one healthy subject from the ABIDE dataset is provided in Figure 5. This visualization corresponds to temporal window *t*_*w*=1_, and the nodes of the network represent the voxels within the left amygdala. As that figure demonstrates, the output of the two attention heads for each ST block is sequentially fed into the next ST block, and the output of the last block is supplied to the prediction block. The final convolution layer of the prediction block generates the predictions *y*_*pred*_, which is a matrix of size *N* × *T*_*τ*_ where *N* is the number of nodes (voxels/regions) and *T*_*τ*_ is the temporal window size. This procedure is applied to every entry within each batch for the model to be trained for each region. (In this case, the model is trained for the left amygdala.) Note that the transformer model can be trained on different spatial resolution levels. In our voxel-level experiments, we trained the model for each region separately in parallel, and then used an ensemble voting criteria for the prediction step. This setup has the benefits of more localized representation learning by considering the biological properties of the regions independently, as well as enhancing the training efficiency.

**Figure 6.**
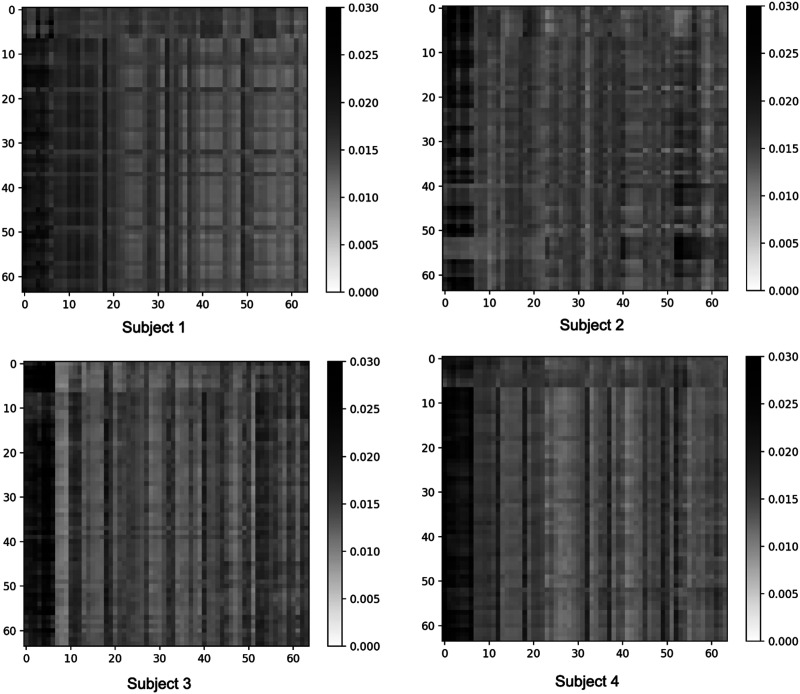
The attention output of the final spatiotemporal (ST) block for the left amygdala of four subjects at temporal window *t*_*w*_ = 2, with 64 voxels and 64 embedding features.

Further visualizations are provided in Figure 6, which shows the attention results of the left amygdala for four control subjects from the ABIDE dataset within the first batch of data for temporal window *t*_*w*=2_. Such representations can assist interpretable analysis of the underlying contextual information in the data.

**Figure 7.**
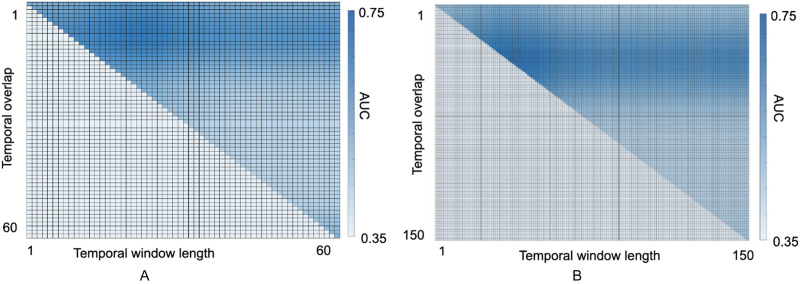
Effect of temporal parameters on AUC. (A) The effect of the length of temporal windows as well as their temporal overlap on average classification AUC for the ABIDE dataset. The values of the cells corresponds to the average classification AUC. Note that the lower triangle does not have any values, as the length of overlap does not exceed the length of the window. (B) The results of the same analysis for the HCP dataset.

Furthermore, the effect of the length of temporal window and the size of the overlap between the windows on classification AUC is provided in Figure 7 for both datasets, where training and testing were performed 10 times on each window-overlap size, and their average AUCs were measured. We can observe that the highest AUCs were achieved on temporal window length and overlap of around 20 and 5, respectively, for the ABIDE dataset, and about 50 and 10 for the HCP dataset. Therefore those window-overlap sizes were adopted for this study. In order to examine and compare the performance of the models with temporal window size, we performed this classification with various lengths of the windows. This analysis is provided in Figure 5 in the Supporting Information, which demonstrates that despite the decline in the AUC, the ST model outperforms the baselines. The decline in AUCs for small window size can be explained by statistically weak and inconsistent functional connectivity information as the length of the time series segments is decreased. On the other hand, the weaker prediction power for large window sizes can be explained by the decrease in the number of time series segments generated as input data, which results in under-training of the model due to small input data size. To further analyze the consistency of attention weights with variations of the temporal window size, we can measure the similarity between the attention matrices. The results of this analysis is provided in Figure 4 in the Supporting Information, where the values of the matrix cells correspond to the similarity between the attention maps measured by mean percentage error (MPE) of the voxel-wise difference (between the values of corresponding matrix cells). Note that the dimensions of attention maps depend on the number of voxels within the regions in voxel-level analysis, therefore they differ from one region to another. We can observe that the attention maps show a strong similarity along the diagonals, meaning that experiments with close temporal window sizes provide similar attention maps, with a slow decline in similarity with the increase in the gap between temporal window sizes across experiments.

**Figure 8.**
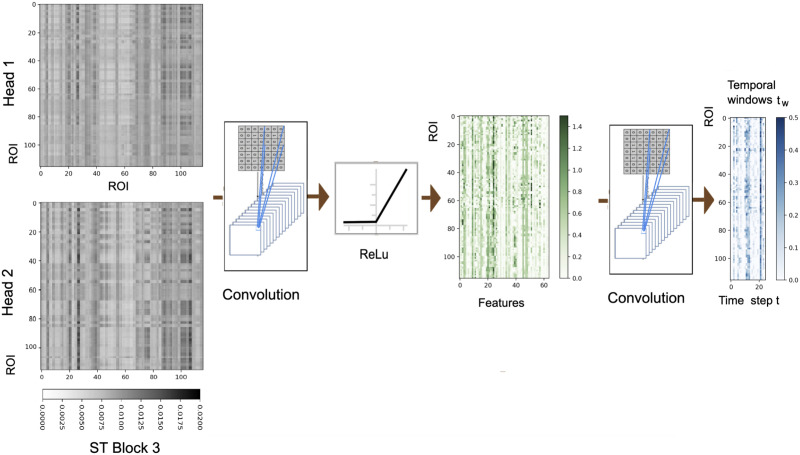
A visualization of the attention map output of each head of the final spatiotemporal (ST) block and prediction block for the full brain setup (116 regions per AAL atlas) of one subject for the first temporal window, where the window size is 25 time steps, and the embedding feature size is 64.

**Figure 9.**
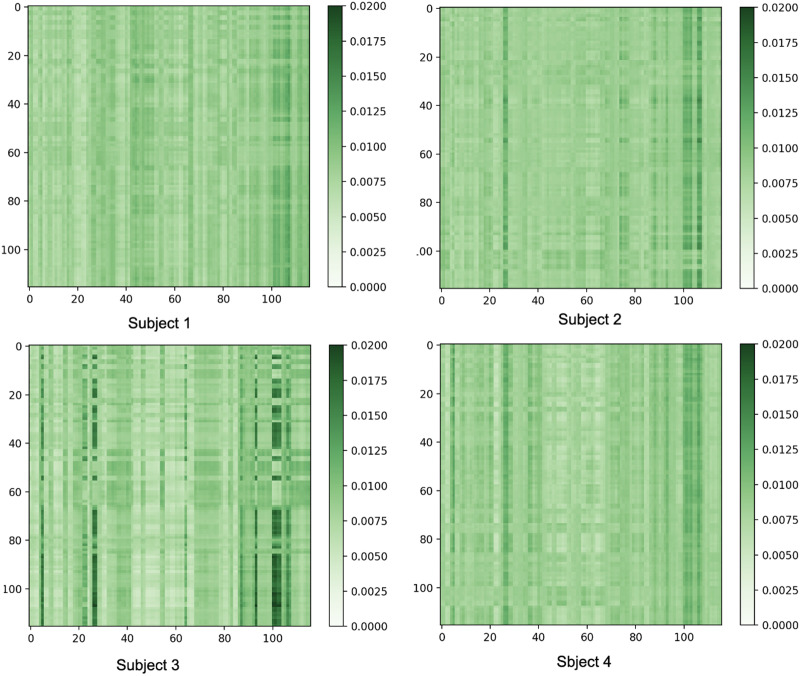
The attention map output of the final spatiotemporal (ST) block for the entire brain of four subjects at temporal window *t*_*w*_ = 2 with 116 ROIs and 64 embedding features.

**Figure 10.**
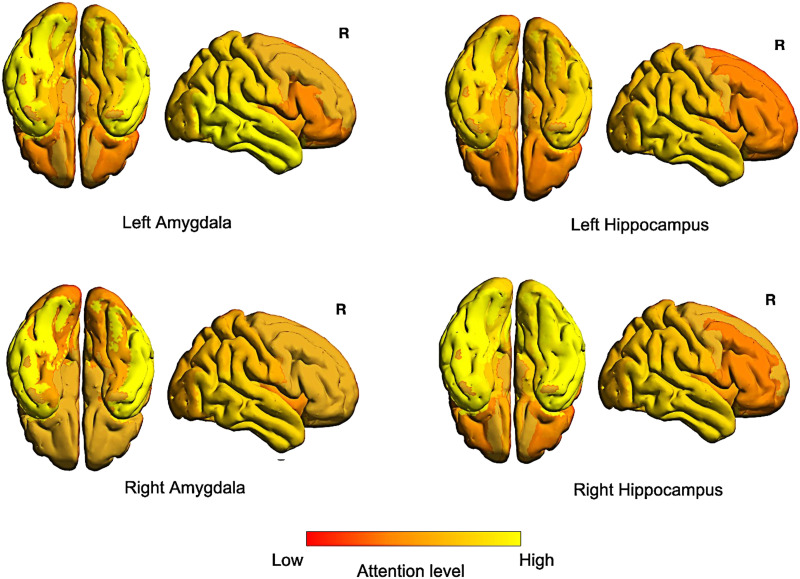
The attention weights of various areas of the brain with regards to the left and right amygdalas and hippocampus, averaged across all healthy subjects in the ABIDE dataset.

For the ROI-level analysis, a visualization of the output of each attention head of the last two ST blocks is illustrated for *t*_*w*=1_ in Figure 8, and the attention outputs for four subjects from the ABIDE dataset are provided in Figure 9. As discussed previously, in ROI-level analysis the nodes of the network correspond to the regions of interest whose fMRI signal is averaged. Also, a visualization of averaged attention weights for 300 healthy subjects based on the ABIDE dataset for the left and right amygdalas and hippocampus is provided in Figure 10. As can be seen in that figure, for the mentioned four regions, we can observe higher overall attention weights for the temporal lobe, and a consistent level of overall attention on the frontal lobe. A similar visualization is provided in the Supporting Information for average attention scores for the second dataset, which demonstrates relatively similar attention patterns. Moreover, visualizations for the attention weight based on four cerebellum regions as the query node are provided in Figure 4 in the Supporting Information. For ease of presentation, we provide the higher attention weights that exceed the top half score cutoff threshold. In that figure we can observe contextual interaction between the cerebellar regions and other cerebellar regions, the amygdalas, and motor and visual cortices. These results can demonstrate the contextual functional interactions between the regions through the framework of attention mechanism. The spatiotemporal attention weights inject this contextual information into the learned representation (features) to assist the prediction tasks.

### Classification Results

**Figure 11.**
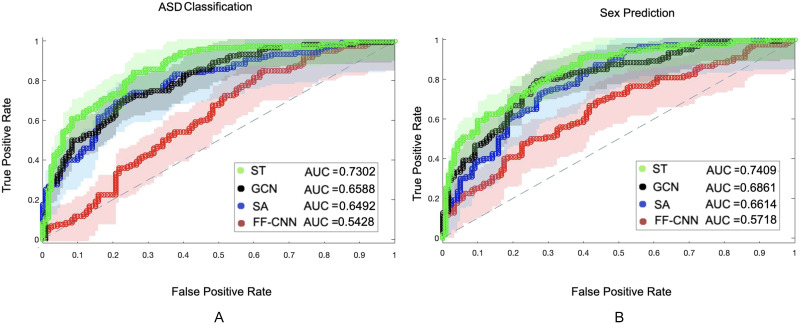
Voxel-level classification results. (A) The voxel-level classification AUC of the ST transformer, graph convolution network (GCN), transformer with only self attention (SA) block, and feed-forward convolution neural network (FF-CNN) for the ABIDE dataset. (B) The classification performance of the same models on the HCP dataset.

The classification results on voxel-level resolution for both datasets is provided in Figure 11, along with the classification confusion matrix in Table 1. As mentioned in the Methodology section, for this analysis a model is trained for each region, and during test a majority voting is performed to provide the final classification. As demonstrated in these results, the spatiotemporal contextual features derived by the ST transformer offer an enhanced pattern extraction compared with the baseline models. In order to provide a more clear analysis of the difference between the AUC values, DeLong’s test for assessing the difference between the AUC values was performed; the null hypothesis is that the true performance of two models are equal. The results of this test are provided in Table 1 of the Supporting Information. As can be seen in that table, the null hypothesis is rejected between the ST method and the baseline methods. This can be explained by the broader information that the features generated by the ST model retain through exploiting the spatiotemporal contexts of BOLD activations as well as the functional connectivity network of the regions during the experiment. In order to evaluate the consistency of classification votes of each region, the percentage of subjects classified as healthy for the ABIDE dataset and the percentage of subjects classified as female for the HCP dataset for every region are provided in Figures 6 and 7 of the Supporting Information. Note that these percentages include false and true positive/negative classifications.

**Table 1.** The confusion matrix for the voxel-level classification based on the spatiotemporal transfromer model based on the ABIDE (left) and HCP (right) datasets

		Predicted ASD		Total			Predicted sex		Total
		Positive	Negative				Female	Male	
True label	Positive	33	12	45	True label	Female	24	9	33
	Negative	11	34	45		Male	7	26	33
	Total	44	46	90		Total	31	35	66

**Figure 12.**
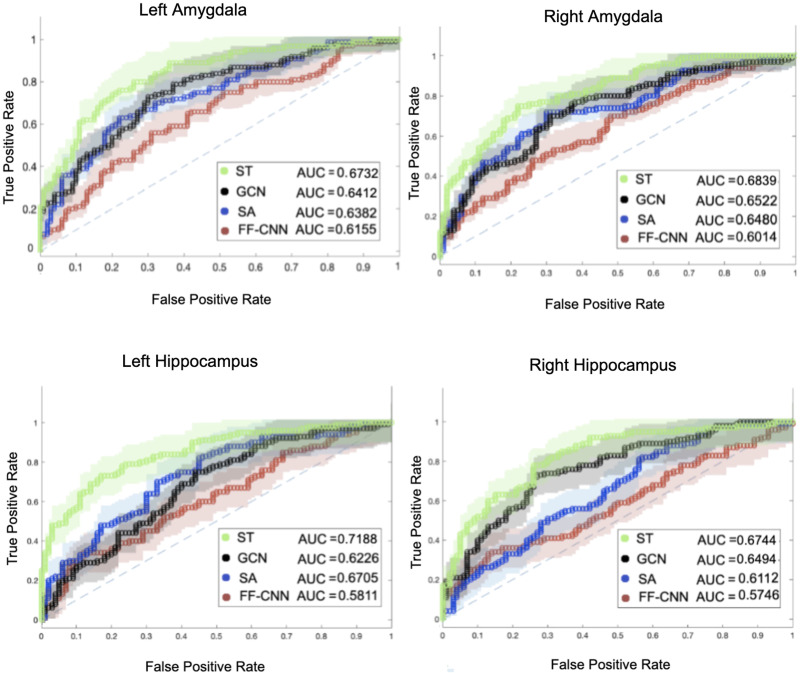
The classification AUC of the ST transformer, graph convolution network (GCN), transformer with only self attention (SA) block, and feed-forward convolution neural network (FF-CNN) for four regions of interest of 600 subjects from the ABIDE dataset.

Moreover, the classification power of separate regions of interest can be examined by training the model on an ROI and calculating the prediction AUC on data of the same region from test subjects. Since the dataset is balanced, we also provide the accuracy for all regions in Table 4 of the Supporting Information. Figure 12 demonstrates the classification performance of the same models on four regions of interest, including the left and right amygdalas and hippocampus from the ABIDE dataset, where the voxels within each ROI constitute the nodes of the FC network. The results of this analysis for the second dataset are provided in Figure 2 in Supporting Information. We can note a decrease in classification performance for training the model on only one region compared with all regions, which was carried out in the previous analysis.

As the last step of our analysis, we set up two ROI-level classification tasks. In order to prepare the input batches for this analysis, we derived the average time series of each region of interest and performed the same segmentation approach as the voxel-level analysis. Therefore, regions of interest were set as the nodes of the FC networks instead of the voxels within the regions, and one training task was performed instead of parallel training on separate regions. Through this process, a dataset size of 15,000 segments was generated for the ABIDE dataset, and 31,680 segments for the HCP sample. The dFC networks were also generated for each temporal window, where the nodes represented regions of interest, and the weights of the links between them were calculated based on the correlation between the average ROI time series within each temporal window *t*_*w*_.

**Figure 13.**
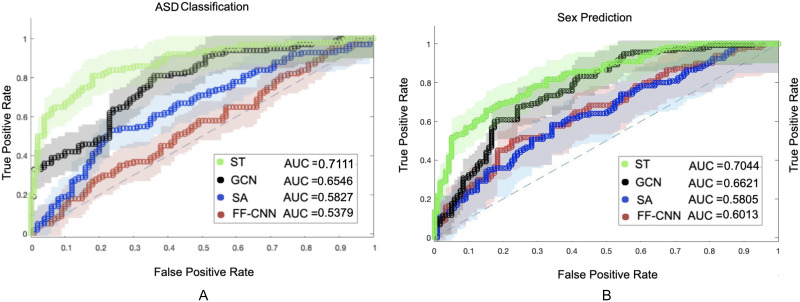
(A) The autism spectrum disorder (ASD) classification AUC of the ST transformer, graph convolution network (GCN), transformer with only the self attention (SA) block, and feed-forward convolution neural network (FF-CNN) on ROI-level setup for 600 subjects from the ABIDE dataset. (B) The classification AUC for sex classification on ROI-level setup for the HCP dataset.

The results of classification tasks based on both datasets are provided in Figure 13 along with the confusion matrix in Table 2, where the same baseline methods as the voxel-level analysis were adopted. As Figure 13 demonstrates, the ST approach provides a more informative representation of the fMRI data compared with the baseline methods. DeLong’s test results are also provided for the ROI classification setup in Table 2 of the Supporting Information, which shows that the null hypothesis is rejected between the ST method and the baseline approaches. However, a drop in the overall classification performances is noticeable compared with the voxel-level analysis in Figure 13. The difference between the results of the voxel-level and ROI-level setups can be explained by the loss of information due to the lower spatial resolution of the input data, which also affects the topology and weights of the dynamic connectivity networks.

**Table 2.** The confusion matrix for the ROI-level classification based on the spatiotemporal transfromer model based on the ABIDE (left) and HCP (right) datasets

		Predicted ASD		Total			Predicted sex		Total
		Positive	Negative				Female	Male	
True label	Positive	33	12	45	True label	Female	22	11	33
	Negative	14	31	45		Male	8	25	33
	Total	47	43	90		Total	30	36	66

### Ablation Analysis

Table 3 shows an ablation study to assess the significance of different architectural blocks on the classification performance of the ST model. For this purpose, we designed two experiments. In the first experiment we excluded three subcomponents of the model, including the positional encoding, the attention block, and the GCN block one at a time. In the second experiment, the entire spatial and temporal blocks were removed separately to assess the model’s performance in their absence. A first observation of the results in Table 3 indicates a level of degradation in the model’s performance with removal of each of its components. This deterioration is more prominent in the second experiment, where one of the spatial or temporal blocks is entirely removed. Also, as we can observe from this analysis, removal of the attention block affected the model’s performance relatively more severely compared with removal of the GCN block. A conclusion one can derive from these two observations is the emphasis on the significance of the process of enhancing the relevant nodes (removal of attention mechanism in the spatial transformer) and time points (removal of temporal transformer that contains the temporal attention) for the classification task while diminishing other regions and time points through the self-attention mechanism. However, including the GCN block in the model provides a superior performance compared with the model with ablated components.

**Table 3.** Ablation analysis. Left: Average ROI-level classification AUC for ablation analysis of the ST transformer over 10 trials. Right: Average ROI-level classification AUC for four deep learning–based models over 10 trials

Model	ABIDE	HCP	Approach	ABIDE	HCP
Without attention	0.626	0.618	ST-GCN	0.677	0.651
Without GCN	0.650	0.663	Deep-fMRI	0.649	0.640
Without spatial	0.581	0.592	MsRNN	0.668	0.654
Without temporal	0.619	0.634	ST	0.711	0.704
Full model	0.711	0.704			

### Comparison With Deep Learning–Based Models

In order to gain further insight about the performance and characteristics of the spatiotemporal transformer model, we compare it with a number of state-of-the art deep learning approaches that are used in fMRI data modeling. Specifically, convolution, graph convolution, and RNN-based approaches have gained significant attention during recent years in the computational neuroscience community because of their robust performance and flexibility in analysis of images, time series data, and graph structured data (Gadgil et al., 2020; Hjelm, Plis, & Calhoun, 2016; Qu, Hu, Xiao, & Wang, 2020; Wang, Li, Chen, & Hu, 2019; Zhao et al., 2018). The general schema of many of such approaches includes a convolution network for obtaining correlations between brain regions and another deep network for the prediction task (Gadgil et al., 2020; Hosseini, Tran, Pompili, Elisevich, & Soltanian-Zadeh, 2020; Huang et al., 2018; Sarraf & Tofighi, 2016a). For this analysis, three baselines are selected, including the spatiotemporal graph convolutional networks (ST-GCN), deep-fMRI, and the multiscale RNN (MsRNN) (Gadgil et al., 2020; Kong et al., 2021; Riaz et al., 2020; Yan et al., 2019; B. Yu et al., 2017).

ST-GCN is a model for learning from graph-structured time series data (Gadgil et al., 2020). In this baseline, the fMRI data are parcellated and normalized and the average ROI signals are supplied into the model as one-channel spatiotemporal features. These data are processed by three layers of spatiotemporal graph convolution that learn the importance of spatial graph edges for the prediction task and supply this information to the prediction layer for classification (Gadgil et al., 2020). Deep-fMRI is an end-to-end deep learning framework that was developed for classification of fMRI data. The inputs to this model are parcellated BOLD signals (Riaz et al., 2020). A convolution network is then used to extract features as a vector for each brain region. Next, a multilayer perceptron (MLP) regression layer operates on each pair of regions to predict a correlation matrix. Finally, the generated matrix is used by an MLP classification layer to produce a prediction for the subject (Riaz et al., 2020). MsRNN is another deep learning–based approach, which mainly consists of two components: a CNN block that is used as an encoder for obtaining correlations between the brain regions, and an RNN block that is utilized for sequence classification. In RNNs the output of a layer is used as input for the layer itself, thus forming a feedback loop. This property allows the RNN to consider a history of the data sequence that can be used for prediction of the next sequence elements.

A comparison of the ST transformer approach and the three mentioned baselines for the ROI-level classification tasks is provided in Table 3. The enhanced performance of the the ST transformer compared with the baseline approaches, as can be observed in Table 3, can be explained by certain advantages of the attention-based spatiotemporal features compared with CNN-based features. An advantage of attention mechanism compared with convolution-based approaches is that in contrast to the CNN where the receptive field is a neighborhood window of the filter, the receptive field for spatial attention is the entire graph, and for temporal attention is the entire time series. This property provides longer range contextual information for each node (and time point) by considering the global information within the data. Another major difference between the attention mechanism and convolution is that once learned, the temporal or spatial CNN kernels are static. In contrast, instead of calculating the dot product of the input region with a set of fixed kernels, the attention query and key matrices are used to dynamically calculate a new set of kernels for each position in the data sequence. The above-mentioned properties can provide new insight about dynamic codependencies not only between regions of the brain but also between the activation patterns of the same region over time. Moreover, because of their capability in determining the most relevant parts of the input sequence for a certain output, transformer architectures can offer a new point of view regarding the importance of certain interactions between regions of the brain and their temporal behavior in performing various tasks.

In principle, the spatiotemporal transformer builds upon the core concepts of convolution and sequence modeling by combining a graph convolution network (in the spatial block) and the attention mechanism as described in the Methodology section. The flexibility and modularity of this architecture also allows for explorations in design of other architectures based on concepts of deep learning to enhance the modeling of neurological conditions or different tasks.

## DISCUSSION

In this paper, we proposed a framework to extract an spatiotemporal representation of the fMRI data by embedding the context of dynamic variations in multivariate BOLD time series and the characteristics of the dFC networks. This framework adopts attention mechanism for learning the contextual dynamic features and graph convolution network to inject the functional connectivity network–based information in the representation learning task. The spatial and temporal units are then used as the building blocks of a sequential spatiotemporal transformer model with residual connections that supply the encoded features to the prediction unit. In order to prepare the input data, a sliding-window segmentation process is applied to generate batches of time series segments as well as functional connectivity networks within each window. Therefore, for each region of interest (or voxel) a set of features are extracted at each time point after the training process, and these features are then used as the inputs to the prediction layer.

By training the model on each region of interest separately on a voxel level, we examined the prediction power of the regions individually. For the ABIDE dataset, we can notice the importance of the amygdalas, insula, hippocampus, inferior frontal gyrus, and cerebellar regions for predicting ASD. Moreover, for the sex classification task for the HCP dataset, the left cingulum posterior (denoted as Cingulum_Post_L in Table 4 of the Supporting Information), right anterior cingulate cortex (Cingulum_Ant_R), left insula, middle temporal gyrus, cerebellum, and hippocampus exhibit a stronger feature importance. These findings are in line with several studies on ASD as well as sex prediction (Chaddad, Desrosiers, Hassan, & Tanougast, 2017; Dhamala, Jamison, Sabuncu, & Kuceyeski, 2020; Heinsfeld, Franco, Craddock, Buchweitz, & Meneguzzi, 2018; Weis et al., 2020; Q. Xu et al., 2020). Moreover, the classification results exhibit a superior performance from the classifier based on the learned features of the proposed framework compared with the baseline approaches. Several other studies used machine learning methods for predicting ASD and sex based on similar or different datasets. The input features used in many of such studies consist of the characteristics of functional connectivity networks or statistical attributes of BOLD time series. Learning contextual representations by jointly leveraging information within the FC network and time series data can offer a set of informative features that enhance our understanding of interactions within (voxel level) and between (ROI level) the regions and model’s prediction power. The proposed approach benefits from several analytical advantages that we discuss in this section, followed by a discussion regarding its limitations, and suggestions for methodological improvements and future work.

### Joint Learning Framework Provides Superior Pattern Separation

Combining the embedding of the information regarding time series dynamics and dFC provides a more powerful set of features for pattern separation tasks compared with adopting only one of the two input structures. Therefore, the two major sources of information in analysis of fMRI data provide a more precise characterization of the higher order dynamics and contexts of the data when embedded jointly.

### Dynamics of the Functional Connectivity Are Included in the Learned Representation

As explained in the Methodology section, the input batch preparation step includes generating the functional connectivity graphs of each entry of each batch to be utilized by the GCN unit of the spatial component. The FC graphs are created for the time series within each temporal window, similar to the commonly performed dFC network creation based on sliding-window segmentation. Therefore, the variations in the functional connectivity weights of the entire dataset are included in the training and feature encoding process (for *N* subjects and *M* time series segments, *N* × *M* connectivity networks are generated). Consequently, the proposed setup takes advantage of the dynamics in the FC network weights as an important source of information regarding functional dependencies during the course of the fMRI experiment.

### Spatial Precision Analysis

The proposed framework displayed enhanced performance in voxel-level experiment compared with the ROI-level setup. While the ROI-level setup provides a significantly more efficient training, it is limited due to loss of information regarding spatial and functional connectivity context. Therefore, for a transformer encoding block, in which the breadth of inferred information is a determining factor in its performance quality, it is favorable to increase the spatial precision of the analysis. Moreover, large models such as transformer architectures commonly show an improved performance with datasets with a high level of granularity, even in the presence of noise confounds, which is an advantageous factor with voxel-level fMRI data analysis.

### Architecture Flexibility and Transfer Learning

The experimental setup for the classification task included using the encoded features as the input to the convolution-based classifier. The set of features created after training the transformer model can be utilized by various classifier models for comparison and exploratory analysis. This is viable because of the flexibility of the transformer framework in being coupled with other models as decoder and prediction or other analytical blocks through the transfer learning paradigm.

### Limitations

Despite the advantageous aspects of the transformer framework, it bears a number of limitations, which we discuss in this section.

The data preparation process involves performing a segmentation to create the batches of data suitable for large models, such as transformers. Therefore, instead of using the entire time series for each region, a fraction of it is provided for each entry of the batch, which can result in loss of information regarding longer term variations and trends. However, as fMRI data become available to the scale of tens of thousands of subjects, this problem can be amended and the entire time series of each region within the region of analysis (an ROI or the entire brain) can be used for each data entry to train complex models.

Positional embedding is an essential step for attention-based models. Extraction of complex temporal dependencies can benefit from prior knowledge during preprocessing to play the role of inductive bias. In this work, we injected the spatial positional embedding using the functional connectivity matrices, and the temporal positional embedding by calculating the trigonometry-based values of the time steps. Exploring other positional embedding approaches can enhance the training of attention weights, and in turn the prediction performance of the model.

Large models such as transformers with attention mechanisms are restricted by large input dataset and memory. Moreover, despite the advantage of transformers over sequential models such as RNN and LSTM due to their ability in parallel training, sequential architecture of the ST blocks coupled with the GCN units within the spatial components decrease the efficiency in the inference step.

As future work, we would like to explore extraction and comparison of the representations with various brain atlases, as well as analysis of the attention-based context maps across functional networks and different datasets.

## ACKNOWLEDGMENTS

This work was supported in part by National Institutes of Health. The content is solely the responsibility of the authors and does not necessarily represent the official views of the National Institutes of Health.

## SUPPORTING INFORMATION

Supporting information for this article is available at https://doi.org/10.1162/netn_a_00281.

## AUTHOR CONTRIBUTIONS

Nima Asadi: Conceptualization; Data curation; Formal analysis; Investigation; Methodology; Software; Visualization; Writing – original draft; Writing – review & editing. Ingrid R. Olson: Investigation; Supervision; Validation. Zoran Obradovic: Formal analysis; Supervision; Validation.

## FUNDING INFORMATION

Ingrid R. Olson, National Institutes of Health, Award ID: 2R56MH091113-11. Ingrid R. Olson, National Institutes of Health, Award ID: R21HD098509. Ingrid R. Olson, National Institutes of Health, Award ID: R01HD099165.

## TECHNICAL TERMS

Data feature: Features are measured properties or characteristics of a phenomenon. The objective of predictive learning such as classification and forecasting is to learn the connection between patterns in these properties with the outcome variables.
Attention: Attention is a mechanism that calculates the weight of each part of the input data to dynamically highlight relevant features. This process allows the model to focus on the more significant part of the data.
Transformer models: A transformer is a deep learning model that uses the mechanism of attention at its core to create an encoder-decoder structure for prediction and modeling tasks.
Graph convolution network: The graph convolution network is a deep learning model for representation learning and prediction on graph-structured data. It is based on the concept of convolution on the neighborhood of each node of the graph.
Positional embedding: Positional embedding (or encoding) injects the positional context into the input data that are then used by the attention layer for extracting the contextual information.
Input batch: Batches are groups of training data (commonly with a fixed size) on which the deep learning model trains. At the end of training on each batch, the predicted values are compared with the expected output variables to calculate an error. From the error, the weight parameters are updated to improve its predictive performance. This process takes place until all training batches are trained on.
Gate mechanism: The gate mechanism is a block of deep learning architecture that is used to fuse the output of multiple blocks together.
Latent features: As opposed to observable features, latent features are the result of more complex dependencies within the data that can be extracted via the encoder block of the transformer models.
Cross-validation: Cross-validation is the process of using a subset of data, outside the training dataset, to obtain an indication of how well the trained model will generalize on unseen data. This step is carried out before prediction on test data.

## Supporting Information

https://github.com/ThisIsNima/Spatio-Temporal-Transformer
