# Supplementary material for: A transformer model for learning spatiotemporal contextual representation in fMRI data
Source: Netw Neurosci. 2023 Jan 1;7(1):22–47. doi: 10.1162/netn_a_00281 (PMC10270708; doi:10.1162/netn_a_00281)
Supplement: Supplementary file 1 [file netn-7-1-22-s001.pdf]

SUPPLEMENTARY MATERIAL

Asadi, N., Olson, I. R. & Obradovic, Z. (2023). Supporting information for "A transformer model for learning spatio-temporal contextual representation in fMRI data." *Network Neuroscience*, *7*(1), 22–47. https://doi.org/10.1162/netn_a_00281

**Approach**	**ST**	**SA**	**GCN**	**FF-CNN**
**ST**	X	0.01	0.01	<0.01
**SA**	0.01	X	0.03	<0.01
**GCN**	0.01	0.03	X	0.01
**FF-CNN**	<0.01	<0.01	0.01	X

**Approach**	**ST**	**SA**	**GCN**	**FF-CNN**
**ST**	X	0.008	0.011	<0.001
**SA**	0.008	X	0.041	<0.001
**GCN**	0.011	0.041	X	<0.001
**FF-CNN**	<0.001	<0.001	<0.001	X

Table 1. p-values of the DeLong test for comparison of voxel-level classification AUC curves between the four approaches. Left: the p-values for the ABIDE dataset. Right: The results for the HCP dataset.

**Approach**	**ST**	**SA**	**GCN**	**FF-CNN**
**ST**	X	0.02	<0.01	<0.01
**SA**	0.02	X	0.02	<0.01
**GCN**	<0.01	0.02	X	0.01
**FF-CNN**	<0.01	<0.01	0.01	X

**Approach**	**ST**	**SA**	**GCN**	**FF-CNN**
**ST**	X	0.01	0.03	<0.001
**SA**	0.01	X	0.05	<0.001
**GCN**	0.03	0.05	X	<0.001
**FF-CNN**	<0.001	<0.001	<0.001	X

Table 2. p-values of the DeLong test for comparison of ROI-level classification AUC curves between the four approaches. Left: the p-values for the ABIDE dataset. Right: The results for the HCP dataset.

	Age (average)	Male	Female	FD (male)	FD (female)
ABIDE	16.7	461	139	0.11	0.10
HCP	28.7	220	220	0.166	0.162

Table 3. Demographic information of the ABIDE and HCP datasets. FD: frame displacement.

Region	AUC (ABIDE)	AUC (HCP)	Region	AUC (ABIDE)	AUC (HCP)	Region	AUC (ABIDE)	AUC (HCP)	Region	AUC (ABIDE)	AUC (HCP)
Precentral_L	61.3853	61.6825	Frontal_Med_Orb_R	67.0669	66.4749	Occipital_Mid_L	45.4925	50.8802	Pallidum_R	67.1555	56.3180
Precentral_R	62.3702	59.2540	Rectus_L	49.9171	52.4850	Occipital_Mid_R	60.4900	72.6534	Thalamus_L	69.3236	64.6870
Frontal_Sup_L	62.5289	66.4390	Rectus_R	60.3156	65.2167	Occipital_Inf_L	45.0725	65.6983	Thalamus_R	61.9416	50.9183
Frontal_Sup_R	63.5563	61.8391	Insula_L	61.2462	70.1088	Occipital_Inf_R	61.6801	64.3149	Heschl_L	46.6705	67.0408
Frontal_Sup_Orb_L	62.3141	57.3719	Insula_R	60.3011	50.8079	Fusiform_L	61.6720	68.4547	Heschl_R	62.6929	63.6705
Frontal_Sup_Orb_R	61.8158	56.8206	Cingulum_Ant_L	59.6575	65.0483	Fusiform_R	47.2120	62.7055	Temporal_Sup_L	67.0084	67.4978
Frontal_Mid_L	55.3588	52.0223	Cingulum_Ant_R	60.2721	70.4412	Postcentral_L	56.9073	71.6207	Temporal_Sup_R	70.5827	58.7943
Frontal_Mid_R	64.8916	54.6586	Cingulum_Mid_L	64.2744	66.5162	Postcentral_R	68.1661	53.5060	Temporal_Pole_Sup_L	65.6032	62.2287
Frontal_Mid_Orb_L	60.7079	69.0002	Cingulum_Mid_R	65.8096	64.9429	Parietal_Sup_L	62.1761	66.4284	Temporal_Pole_Sup_R	50.0171	57.1032
Frontal_Mid_Orb_R	55.9145	62.7402	Cingulum_Post_L	58.4232	71.5387	Parietal_Sup_R	57.0921	64.8248	Temporal_Mid_L	65.3899	56.6493
Frontal_Inf_Oper_L	68.4869	66.1156	Cingulum_Post_R	64.0909	64.2791	Parietal_Inf_L	60.6997	62.8342	Temporal_Mid_R	67.3871	69.1232
Frontal_Inf_Oper_R	54.8497	62.9362	Hippocampus_L	71.3240	71.3062	Parietal_Inf_R	56.7746	56.1155	Temporal_Pole_Mid_L	61.8301	64.6095
Frontal_Inf_Tri_L	49.7012	67.5399	Hippocampus_R	68.1844	71.1403	SupraMarginal_L	57.0108	56.3376	Temporal_Pole_Mid_R	58.7713	63.4639
Frontal_Inf_Tri_R	67.1845	51.1269	ParaHippocampal_L	60.8891	61.8386	SupraMarginal_R	68.3556	72.9054	Temporal_Inf_L	64.9520	64.7669
Frontal_Inf_Orb_L	66.9521	71.2563	ParaHippocampal_R	70.6511	59.7008	Angular_L	67.9293	66.3002	Temporal_Inf_R	58.0234	69.2289
Frontal_Inf_Orb_R	62.9971	68.8155	Amygdala_L	68.1103	66.5109	Angular_R	64.1646	56.7625	Cerebellum_Crus1_L	65.9211	64.6525
Rolandic_Oper_L	52.2169	54.1218	Amygdala_R	67.1432	67.1472	Precuneus_L	63.4339	62.8657	Cerebellum_Crus1_R	68.2723	69.7713
Rolandic_Oper_R	53.4704	62.5641	Calcarine_L	58.1299	73.0792	Precuneus_R	62.6661	62.0263	Cerebellum_Crus2_L	63.0120	63.7231
Supp_Motor_Area_L	68.9621	63.2955	Calcarine_R	62.0085	71.8029	Paracentral_Lobule_L	63.4989	51.7043	Cerebellum_Crus2_R	70.3115	51.9105
Supp_Motor_Area_R	62.6083	65.6001	Cuneus_L	62.4675	64.0841	Paracentral_Lobule_R	52.1933	59.7211	Cerebellum_3_L	62.8065	61.8579
Olfactory_L	54.0526	64.1142	Cuneus_R	54.4829	57.4190	Caudate_L	68.7325	70.1301	Cerebellum_3_R	64.0489	71.2432
Olfactory_R	53.8834	64.2294	Lingual_L	53.4307	67.7510	Caudate_R	66.1694	69.6664	Cerebellum_4_5_L	69.7780	60.0770
Frontal_Sup_Medial_L	51.7562	64.4418	Lingual_R	63.5670	72.5274	Putamen_L	67.1363	52.9994	Cerebellum_4_5_R	72.4775	55.9199
Frontal_Sup_Medial_R	60.1633	65.4372	Occipital_Sup_L	51.6133	71.8550	Putamen_R	49.1525	60.2994	Cerebellum_6_L	53.0206	63.5372
Frontal_Med_Orb_L	61.9511	58.5390	Occipital_Sup_R	50.1605	70.7150	Pallidum_L	45.1647	69.9517	Cerebellum_6_R	53.8805	59.2227
Cerebellum_7b_L	68.9630	58.5754	Cerebellum_8_R	74.1863	64.0556	Cerebellum_10_L	52.2764	55.5045	midline Vermis_3	52.0546	65.9616
Cerebellum_7b_R	69.2259	72.3711	Cerebellum_9_L	58.8952	69.7336	Cerebellum_10_R	57.8309	70.5708	midline Vermis_4_5	57.7429	55.2035
Cerebellum_8_L	58.6577	62.6040	Cerebellum_9_R	54.8299	58.7732	midline Vermis_1_2	63.9586	51.9010	midline Vermis_6	71.3874	58.3281

Table 4. Average classification AUC for all of the regions of interest (per AAL atlas) based on the ST method on the ABIDE and HCP datasets .

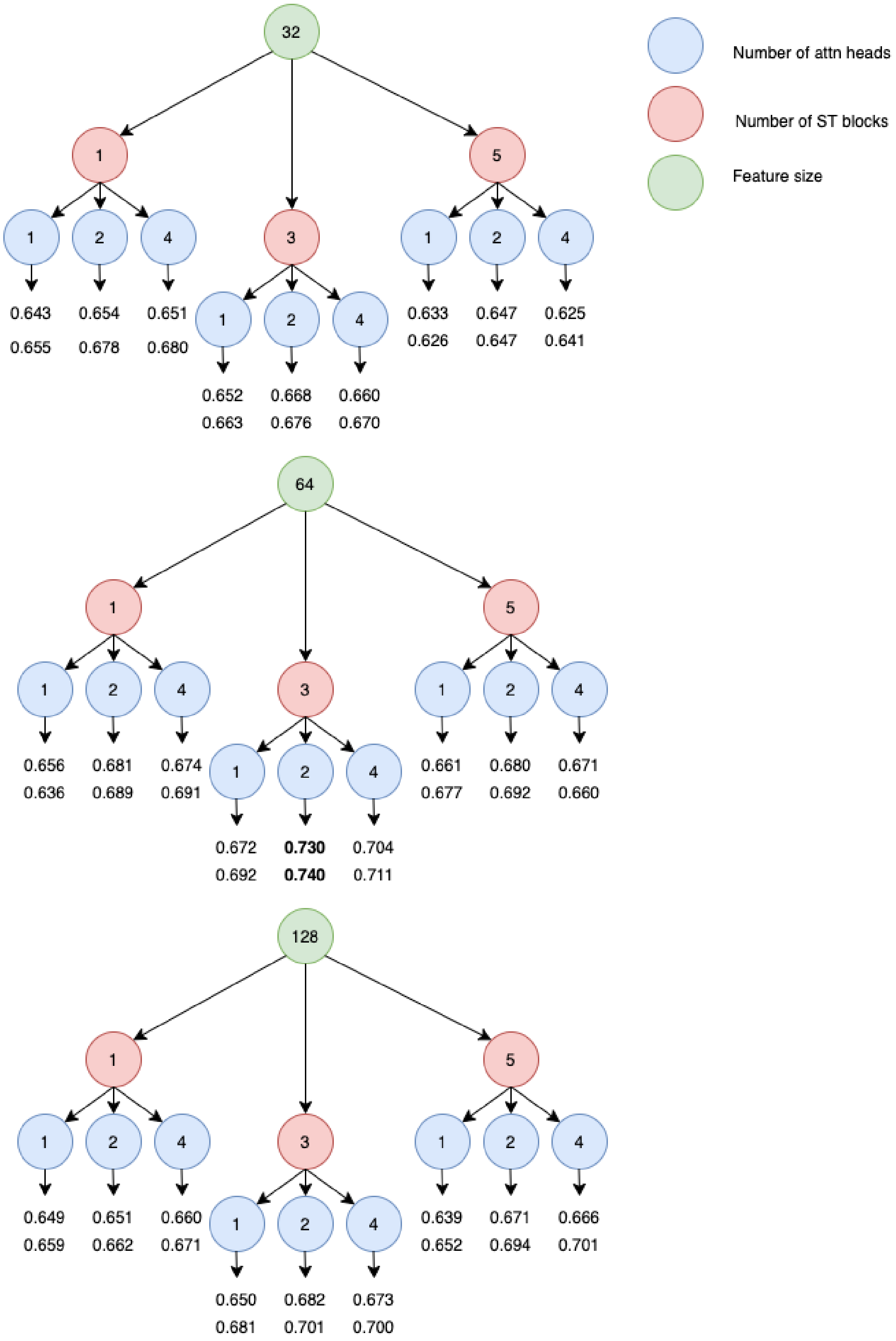

Figure 1. The average classification AUC of the model based on different architectural configurations for both dataset. The top AUC values correspond to the ABIDE dataset, and the bottom values correspond to the average AUC on the HCP data.

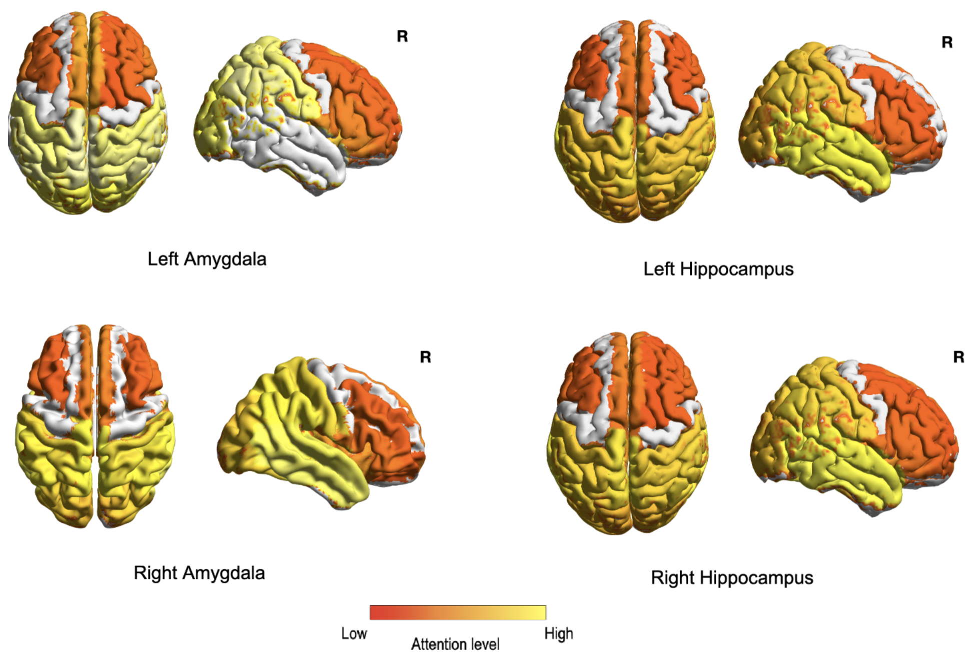

Figure 2. Visualization of average attention weights of various regions of the brain with regards to the left and right Amygdalas and Hippocampus among all subjects from the HCP sample.

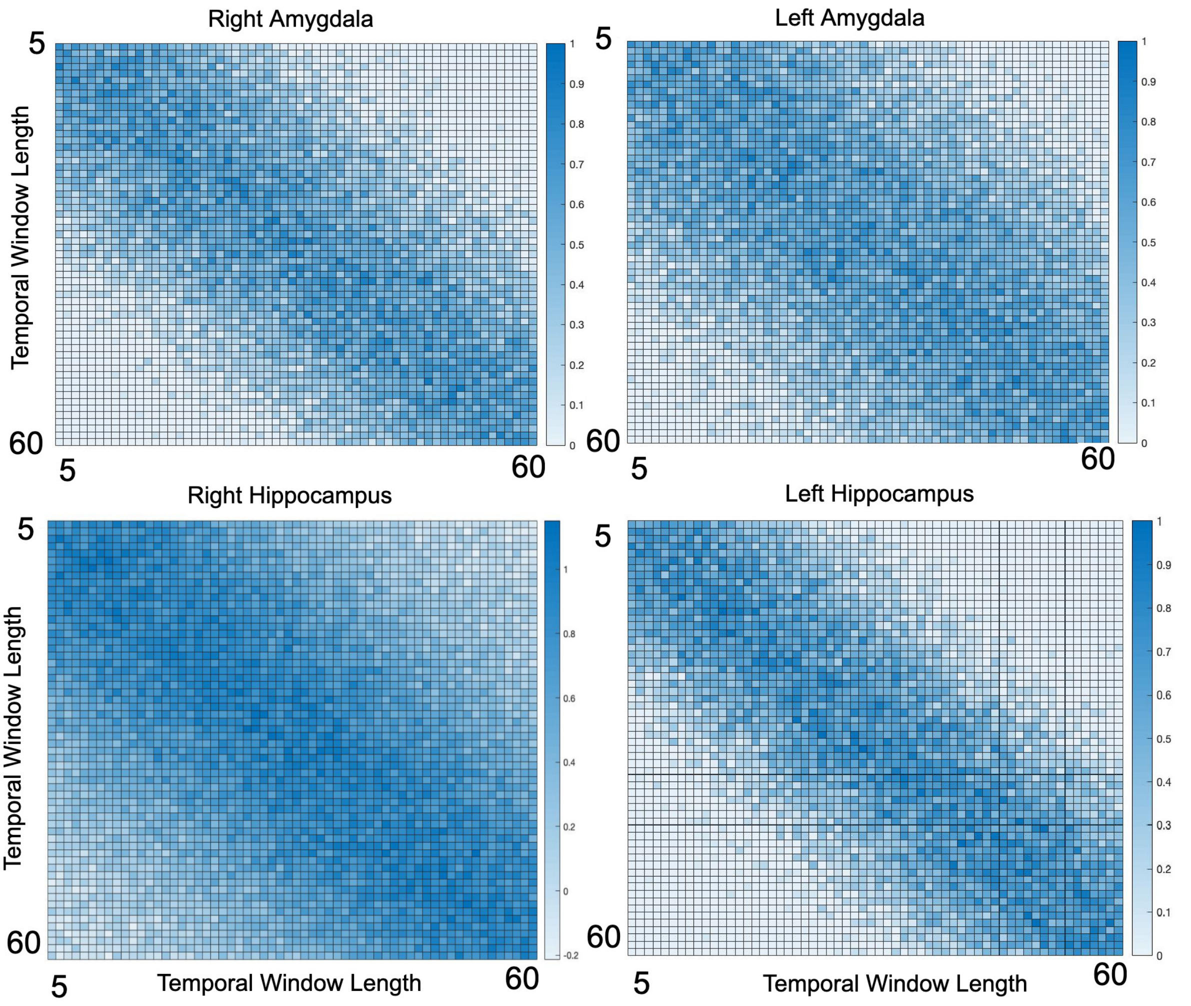

Figure 3. The effect of temporal window length on attention maps. The values of the matrix are the similarity between the attention maps

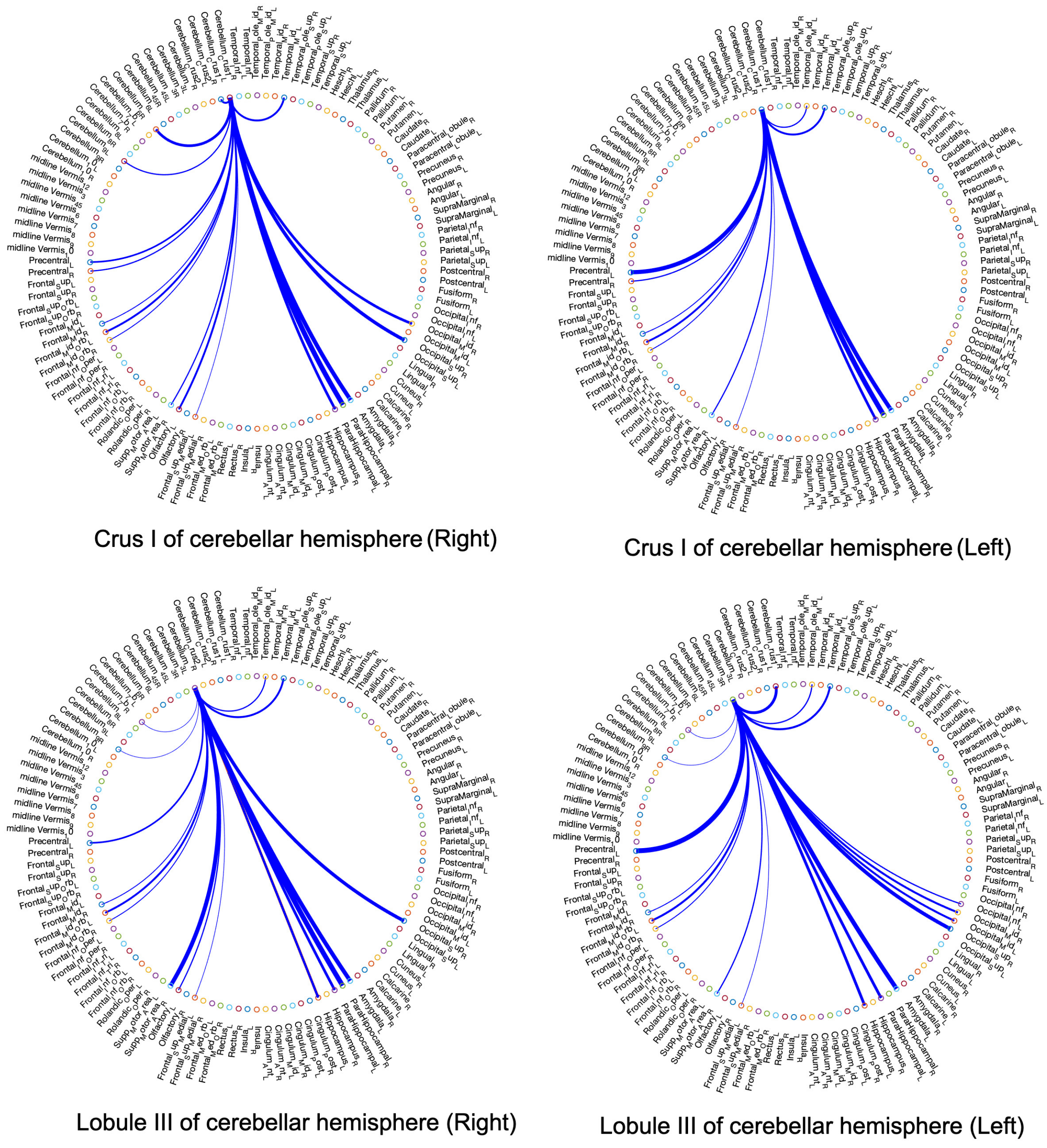

Figure 4. The attention weights for four cerebellar regions as the query nodes. The width of links correspond to higher attention similarity and contextual interaction between the regions.

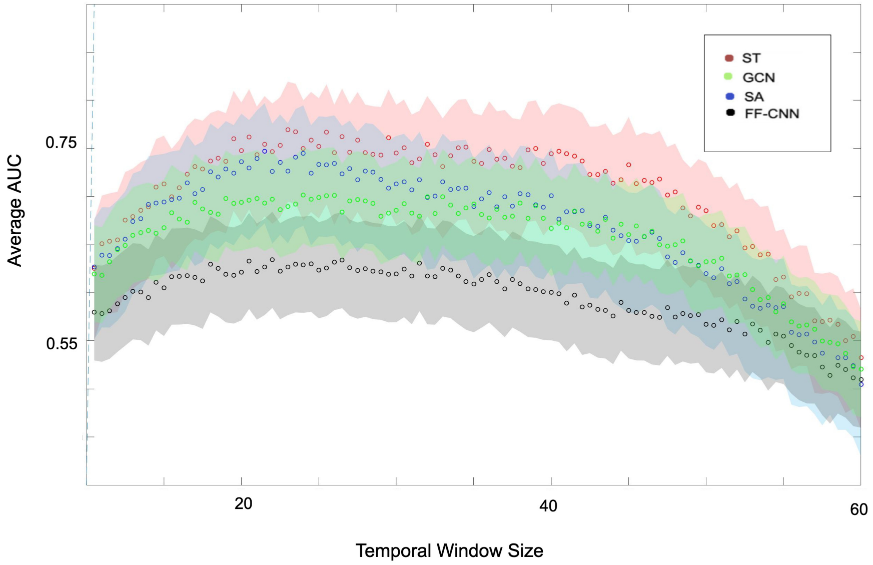

17 **Figure 5.** The effect of temporal window length on average AUC of the four models.

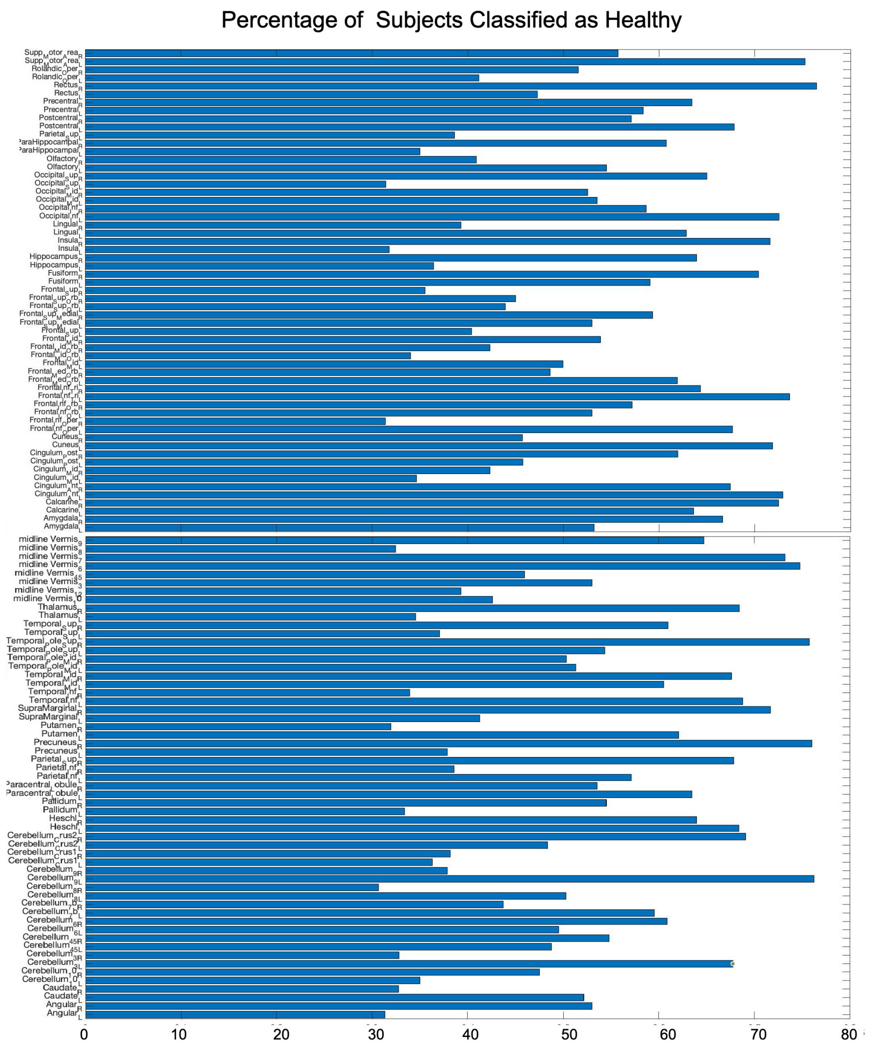

Figure 6. Percentage of subjects classified as healthy from each region for the ABIDE dataset.

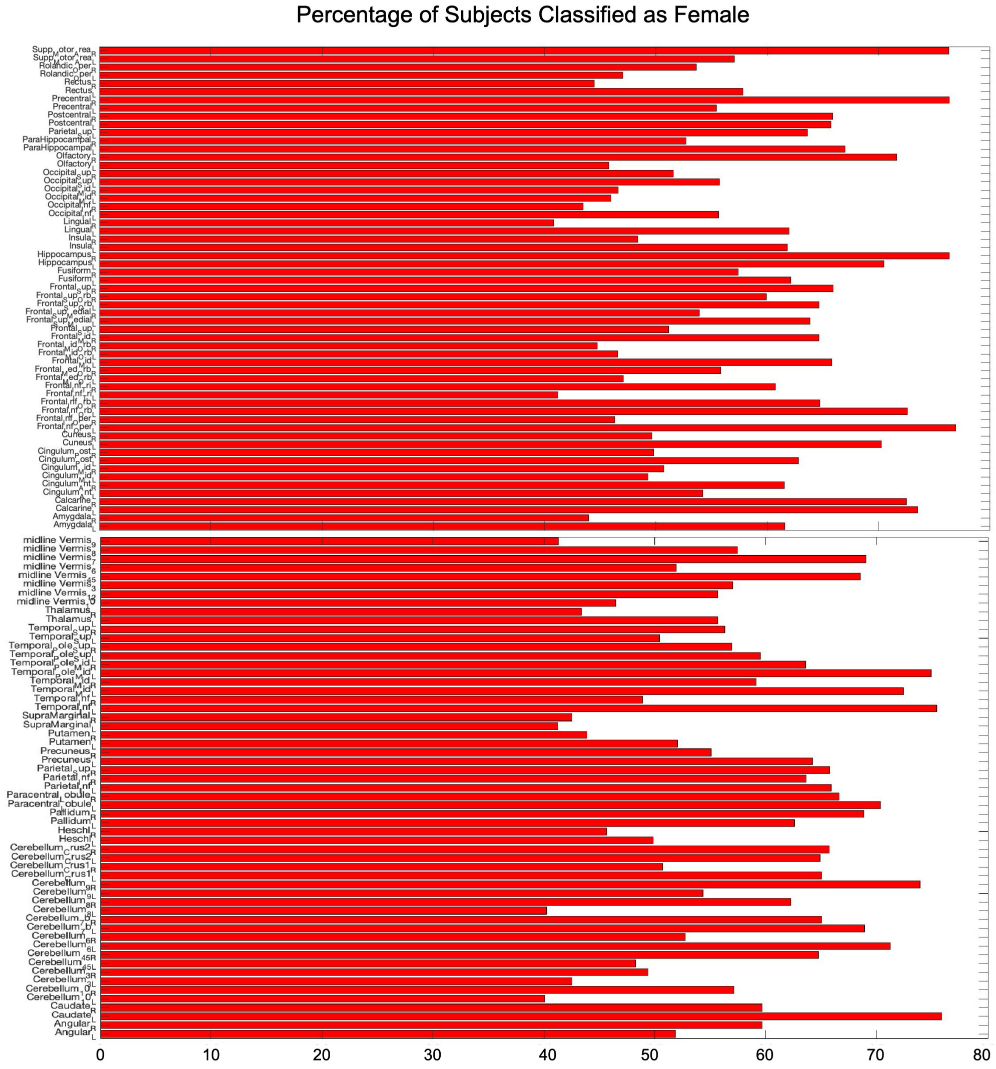

Figure 7. Percentage of subjects classified as female from each region for the HCP dataset.
